# Contrast Agents Delivery: An Up-to-Date Review of Nanodiagnostics in Neuroimaging

**DOI:** 10.3390/nano9040542

**Published:** 2019-04-03

**Authors:** Daniel Mihai Teleanu, Cristina Chircov, Alexandru Mihai Grumezescu, Adrian Volceanov, Raluca Ioana Teleanu

**Affiliations:** 1 Emergency University Hospital, “Carol Davila” University of Medicine and Pharmacy, 050474 Bucharest, Romania; daniel.teleanu@umfcd.ro; 2 Faculty of Engineering in Foreign Languages, Politehnica University of Bucharest, 060042 Bucharest, Romania; cristina.chircov@yahoo.com; 3 Faculty of Applied Chemistry and Materials Science, Politehnica University of Bucharest, 011061 Bucharest, Romania; grumezescu@yahoo.com; 4 ICUB—Research Institute of University of Bucharest, University of Bucharest, 36-46 M. Kogalniceanu Blvd., Bucharest 050107, Romania; 5 “Victor Gomoiu” Clinical Children’s Hospital, “Carol Davila” University of Medicine and Pharmacy, 050474 Bucharest, Romania; raluca.teleanu@umfcd.ro

**Keywords:** neuroimaging, neuroscience, nanotechnology, imaging, contrast agents

## Abstract

Neuroimaging is a highly important field of neuroscience, with direct implications for the early diagnosis and progression monitoring of brain-associated diseases. Neuroimaging techniques are categorized into structural, functional and molecular neuroimaging, each possessing advantages and disadvantages in terms of resolution, invasiveness, toxicity of contrast agents and costs. Nanotechnology-based approaches for neuroimaging mostly involve the development of nanocarriers for incorporating contrast agents or the use of nanomaterials as imaging agents. Inorganic and organic nanoparticles, liposomes, micelles, nanobodies and quantum dots are some of the most studied candidates for the delivery of contrast agents for neuroimaging. This paper focuses on describing the conventional modalities used for imaging and the applications of nanotechnology for developing novel strategies for neuroimaging. The aim is to highlight the roles of nanocarriers for enhancing and/or overcome the limitations associated with the most commonly utilized neuroimaging modalities. For future directions, several techniques that could benefit from the increased contrast induced by using imaging probes are presented.

## 1. Introduction

Affecting approximately a billion individuals worldwide, neurological diseases are one of the world’s leading causes of death. Their prevalence is expected to further increase in the following decade due to the rapid growth in elderly population. Considering the complexity of the brain, the pathogenesis of the central nervous system diseases is not fully understood and therefore the diagnosis and treatment pose serious challenges [1,2].

The clinical manifestations of the most prevalent brain disorders—including neurodegenerative conditions, brain tumours, stroke, traumatic brain injuries, epilepsy and infections—commonly involve dementia, hypokinetic and hyperkinetic movement disorders [3], headaches, seizures, cognitive changes, incontinence, gait disorders [4], depression, cognitive impairments, physical disability [5], fatigue, sleep disorders [6], anxiety [7], nausea, vomiting, photophobia, fever and loss of consciousness [8]. However, to appropriately diagnose brain diseases, the basic neurological examination which includes patient’s history and physical examination is not sufficient. Therefore, identification and usage of proper imaging techniques based on clinical symptoms are crucial for neurologic diagnosis and initial and subsequent management [9,10]. Apart from brain diseases diagnosis, neuroimaging techniques are extensively applied in neuroscience to visualize neural activity, understand brain mechanisms and identify biomarkers [11]. Furthermore, proper biomarkers and neuroimaging techniques are essentially necessary in diagnosis owing to the possibility of early detection of pre-symptomatic pathological changes, efficient differentiation between related neurological diseases, identification of disease progression and treatment effects, non-invasive confirmation of the underlying pathology and screening large populations for risk assessments [12]. Additionally, through the integration of genomic and neuroimaging data, the investigation of genetic risk factors shaping variations in brain phenotypes and mechanisms underlying neurological and neuropsychiatric disorders is possible [13].

The recent advances in neuroimaging have enabled an improved and detailed morphological and functional evaluation of the brain [14]. Depending on the requirements, neuroimaging techniques can be structural, including computed tomography (CT) and magnetic resonance imaging (MRI), which are used for the diagnosis of gross intracranial diseases, such as tumours, strokes, injuries [15] and neurodegenerative diseases [16], functional, including optical imaging, functional magnetic resonance imaging (fMRI), electroencephalography (EEG), magnetoencephalography (MEG), functional near-infrared spectroscopic imaging (fNIRS) and functional ultrasound (fUS) and molecular, including positron emission tomography (PET), single photon emission computed tomography (SPECT) and molecular magnetic resonance imaging (mMRI), each technique providing different information [17,18].

Conventional neuroimaging techniques are characterized by different advantages and disadvantages. Several advantages include high sensitivity for PET and non-invasiveness and cost-effectiveness for optical imaging. However, disadvantages such as low sensitivity, soft tissue contrast and signal-to-noise ratio for CT [19], long acquisition time for MRI, low resolution and short half-life of isotopes for PET and strong scattering of light in biological tissues for optical imaging must be addressed. Considering the limitations of current neuroimaging techniques, there is an emerging necessity for developing novel approaches [20]. Nanotechnology, the continuously evolving field that encompasses knowledge from multiple disciplines including chemistry, physics, engineering and biology, represents a potential strategy to overcome these limitations [21,22]. Nanotechnology implicates the development and modification of materials within the size range of 1–100 nm in at least one dimension, offering the possibility to understand, manipulate and control the matter at the level of individual atoms and molecules [23,24]. Therefore, the implication of nanotechnology to design nanostructures as contrast agents might facilitate imaging for the accurate characterization and resection of tumours, the delivery of therapeutic stem cells and the detection of neurodegenerative diseases in early stages. Furthermore, the nanotechnology-based contrast agents offer high chemical and biological stability and possibilities of multi-functionalization for targeted delivery, administration through various routes and blood-brain barrier penetration [20]. The development of nanostructured contrast agents has also offered the prospect of simultaneously acquiring images using several imaging techniques or capturing images at different moments. Thus, the platform for multimodal imaging allows for benefiting from the advantages of each neuroimaging technique while overcoming their specific limitations [25].

The present article focuses on reviewing the common nanotechnology-based neuroimaging techniques, along with the most recent advancements in the applications of nanotechnology to design novel contrast agents to improve the examination and diagnosis of brain-associated disorders.

## 2. Neuroimaging Techniques

**Figure 1:**
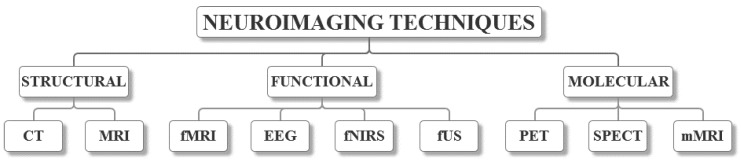
The classification of commonly applied neuroimaging techniques.

Depending on the requirements for the brain images, neuroimaging techniques can be categorized into structural, functional and molecular neuroimaging, each method described, as follows (Figure 1). The characteristics of each method that were found in the literature [26,27,28,29] are summarized in Table 1. However, there have been some issues associated with collecting the information due to the inconsistency between different papers.

### 2.1. Structural Neuroimaging

Structural neuroimaging has almost entirely replaced the conventional studies of the anatomy and morphology of the brain. The clinical applications of structural neuroimaging target the exact diagnosis followed by the individualized treatment plan, whereas in research, it has led to the understanding of the neuroanatomy at the individual and group level. Furthermore, the structural imaging-based measurements of specific lesions has allowed for the association between the symptom severity, lesion load and lesion location. The most common methods involve CT and MRI [30].

#### 2.1.1. Computed Tomography (CT)

The use of CT for the diagnosis of intracranial pathology has replaced common techniques, including radiography, encephalography or even angiography [31]. X-ray beam technologies are responsible for creating tomographic slices of the brain, which result in superior contrast resolution that allows for the distinction between fluids and soft tissues. Additionally, the problem of superimposition that radiographs pose is eliminated [32]. Modern scanners spin or surround the patient, thus imaging the entire volume of tissue. The acquired information is reconstructed into two-dimensional greyscale images of the slices, which represent the maps of tissue density [31]. The main challenge of CT is represented by the compromise of using a low radiation dose that results in lower signal-to-noise and contrast-to-noise ratios and limited discrimination between tissue with slightly different x-ray opacities [33].

Contrast agents might be injected in the body for perfusion CT imaging, which will provide high-contrast perfusion maps for the diagnosis of ischemic stroke [34]. Moreover, this technique is characterized by several benefits, including the increased sensitivity and specificity towards an exact diagnosis and the provision of information regarding prognosis and treatment decisions. However, the increased radiation exposure and imaging time and the administration of toxic contrast agents which could lead to kidney failure are limitations that must be overcome [35]. Additionally, as common contrast agents cannot pass through the blood-brain barrier, novel strategies for the formulation of nanoagents with limited toxicity are necessary [34].

#### 2.1.2. Magnetic Resonance Imaging (MRI)

The standard tool in clinical diagnosis, disease follow up, treatment evaluation and brain development monitoring is the MRI technique. It has been extensively used owing to its non-invasiveness, high-resolution imaging, enhanced contrast between tissues and multiplanar imaging capabilities [36,37]. MRI acquisition is based on the interaction between an external magnetic field and the magnetic moment of the water protons, which causes alterations in the orientation of the spinning nucleus [38]. Structural MRI techniques, including T1- and T2-weighted imaging, diffusion tensor imaging, magnetization transfer imaging and iron sensitive MRI, such as susceptibility weighted imaging and quantitative susceptibility mapping, are used to highlight specific markers for brain diseases [39].

However, this technique is associated with long imaging times as time plays a key role in the quality of the acquired images. Specifically, the longer the acquisition time, the higher the signal-to-noise and contrast-to-noise ratios, as they are dependent on the number of pulse sequences performed [33,40]. Another option to improve the sensitivity and the signal-to-noise ratio is represented by the increase of the magnetic field strength using stronger magnets. However, this strategy involves higher costs and special requirements for installation, which limit the use of magnets with field strengths between 7 T and 21 T [41]. Therefore, to overcome the long imaging time and the high costs associated, the quality of the images must be reduced [33].

### 2.2. Functional Neuroimaging

Functional neuroimaging is applied in brain function evaluation for an improved understanding of the correlations between the activity in specific brain areas and the mental functions [42]. The invention of the blood oxygen level-dependent (BOLD) fMRI has subsequently led to the tremendous advances in the field of functional neuroimaging [43]. Furthermore, the development of multichannel EEG and intracranial electroencephalography (iEEG) and fNIRS has allowed for the understanding of complex anatomical correlations and functional relationships between the lesion and the adjacent cortical areas and white matter structures [42].

#### 2.2.1. Functional Magnetic Resonance Imaging (fMRI)

fMRI has been developed for the study of vascular or metabolic reactions of the brain in response to different stimuli [44]. BOLD fMRI is the leading technique for functional neuroimaging [45]. The acquired signals represent the relative concentrations of diamagnetic oxyhaemoglobin and paramagnetic deoxyhaemoglobin, which rely on the modifications in the vasculature blood oxygenation correlated to neuronal activity, a process called neurovascular coupling [46]. Furthermore, BOLD fMRI reflects the increase in cerebral metabolic rate of oxygen consumption, cerebral blood flow and cerebral blood volume caused by neural activity [45,46]. Specifically, this technique images the dynamic changes caused by neural metabolism changes due to the variations in the oxygenation levels of brain tissue as a consequence to neural responses to specific cognitive processes or spontaneous fluctuations in resting states [47]. Therefore, the signal is different in patients at rest and during cognitive and sensation activity [46,48,49].

Functional magnetic resonance has greatly contributed to the understanding of the impact of brain disorders on the cognitive functions of patients. Therefore, the differentiation between the vegetative state and the minimally conscious state is a very practical clinical application of this technique [50]. However, the low temporal resolution and signal-to-noise ratio are major issues that pose challenges in fMRI measurements [51]. To improve the specificity and sensitivity of the acquired signals, the administration of contrast agents that typically contain gadolinium might be a solution. However, the associated instability, blood-brain barrier permeation difficulties and the toxicity of these agents must be addressed [52,53].

#### 2.2.2. Electroencephalography (EEG)

EEG is a non-invasive neuroimaging technique that records the electrical activity of the brain [54]. Owing to its simplicity and cost effectiveness, EEG is an extensively used technique in neuroscience, providing high temporal resolution and understanding of the underlying mechanisms that generate the spontaneous electrical activity [55]. The signals originate from the ionic movement through the extracellular space of the neurons and therefore the electroencephalogram represents the sum of all synchronous activity of the neurons that have the same approximate vertical orientation to the scalp [54,56]. The signal is measured by placing a sensor or a headset provided with several electrodes on the scalp [54].

Novel strategies involve the implantation of electrodes in the subdural or deep areas. The iEEG, including electrocorticography and stereo-EEG, provides a neuroelectrophysiological signal that offers more accurate information regarding epileptic discharge patterns and diagnostic information that could aid surgical interventions [57,58]. Moreover, it is a neuroimaging technique that could offer an insight into brain activities as it provides high spatial and temporal resolution for the affected brain areas [57]. However, there are several limitations that must be considered, including the accessibility, as it requires specially trained teams of clinicians and investigators and special equipment [59]. Nonetheless, a sufficient number of electrodes must be placed in order to appropriately define the brain network [58]. These electrodes are usually 5–10 mm apart and consequently global coverage of the brain is difficult to achieve. Additionally, as the electrodes used are usually either cylindrical with a contact length of 2 mm, a diameter of 1 mm and a total surface area of 10 mm^2^ or circular plates with a diameter of 2 mm and a surface area of 4 mm^2^, they capture the signal from a large population of cells [59]. However, this approach does not record from sulcal depths, thus it only provides information from the surface of the cerebral crests [60].

Therefore, nanotechnology might offer the solution to improve the accuracy of iEEG method. Nanotechnology has been applied for the development of nanoelectrodes that could be invasively implanted in the brain for a high-quality neural recording and stimulation. Hence, the potential of the next generation of neuroprostheses could lead to the ability to both control the output of the prosthesis by executing a certain motor function and to record relevant sensory information [61]. Several strategies have been employed to design neural probes, including polypyrrole/graphene oxide composite films [62] and polyimide-based probes [63] with platinum and gold electrodes, respectively. However, the available information is limited and further studies are required for designing reliable nanotechnology-based approaches for neuroimaging through iEEG.

#### 2.2.3. Functional Near-Infrared Spectroscopic Imaging (fNIRS)

fNIRS is a promising neuroimaging method which allows for non-invasive and long-term brain function mapping through the measurement and imaging of local changes in haemoglobin concentrations [64]. The emitted near-infrared light onto the scalp is partly absorbed by haemoglobin and partly scattered and collected by specific sensors. The changes in oxygenated haemoglobin and deoxygenated haemoglobin are regarded as an indicator for variations in the regional cerebral blood volume [65]. The study of brain function through fNIRS requires a good acquaintance with how the diffuse optical neuroimage encodes the information related to it [66].

fNIRS presents a series of advantages, including portability, easy to use, reduced costs, robustness to head movement, high temporal resolution and spatial resolution higher than EEG but lower than fMRI [67]. Nevertheless, the disadvantages of this method, including the limited brain regions that can be reached, specifically the cortical regions beneath the scalp and the interference of superficial veins and arteries and extracortical components that limit signal specificity [68].

#### 2.2.4. Ultrasound-Based Functional Imaging Techniques

fUS is a recently developed functional neuroimaging technique which relies on ultrafast imaging scanners. fUS has brought novel insights in neurodiagnostic imaging and blood flow imaging as it can capture over 20,000 frames per second, contrary to the 50 frames per second in conventional ultrasound scanners [69,70]. The principle of fUS is based on the neurovascular coupling, which correlates local neural activity and relative changes in the cerebral blood volume [71]. While BOLD fMRI depends on blood oxygenation, cerebral blood volume is related to the number of red blood cells within a pixel. Furthermore, fUS allows for brain mapping in response to sensory, motor and odour-evoked stimuli [70]. Similarly, the functional transcranial Doppler ultrasound is a non-invasive neuroimaging technique based on blood-flow velocities within cerebral arteries measurements. It is a low cost, simple and safe imaging modality with high temporal resolution and accuracy, that measures brain activity and functional lateralization [72].

Another ultrasound-based functional neuroimaging technique is photoacoustic tomography, which is used for functional, metabolic and histologic imaging through endogenous contrast and for molecular and cellular imaging through exogenous contrast [73]. Photoacoustic computed tomography (PACT) uses diffused high energy pulsed laser light to illuminate the tissue, which will subsequently generate photoacoustic waves. Deeper imaging depths are usually targeted with PACT but with lower spatial resolution [74]. Based on the detection geometry, PACT systems can be categorized into circular-view PACT, which provides cross-sectional brain images, planar-view PACT, which detects photoacoustic signals along a two-dimensional plane and spherical-view PACT, which is ideal for volumetric imaging, as it provides nearly isotropic spatial resolution [75].

### 2.3. Molecular Neuroimaging

Visualizations, measurements and understanding of biochemical processes and mechanisms at the molecular and cellular levels of the brain have been possible owing to the advances in the non-invasive molecular neuroimaging techniques. Common molecular imaging modalities involve PET, SPECT [18] and mMRI.

#### 2.3.1. Positron Emission Tomography (PET)

PET is a minimally invasive imaging procedure, extensively applied in the evaluation of the neurophysiology of the normal brain and the pathophysiology of various brain disorders [76]. Images are acquired through the internal administration of nanomolar quantities of target-specific radiopharmaceuticals. A camera is used to detect two coincident high energy gamma-rays resulting from the annihilation of the emitted positron with a nearby electron [77]. Although the most common clinical radiopharmaceutical is ^18^F-fluorodeoxyglucose, which quantifies the rate of glucose metabolism, there are countless other tracers which study different molecular processes, including amino acid metabolism, blood flow and neurotransmitter systems [78]. Moreover, by assessing the activity at the serotonin and dopamine receptors, the mechanisms underlying anxiety, depression and addiction can be studied [79].

PET is characterized by a spatial resolution similar to fNIRS, specifically lower than fMRI and EEG and a low temporal resolution. Furthermore, as it requires the injection of a radioactive tracer, the number of measurements performed on the patient is limited [79].

#### 2.3.2. Single Photon Emission Computed Tomography (SPECT)

SPECT is a molecular imaging technique that is commonly used for the diagnosis and therapy monitoring of brain diseases [80]. Neuroimages are acquired through the detection of a photon emitted by a single photon-emitting radionuclide during its radioactive decay. The gamma-ray energy is converted into light in the dedicated gamma-camera crystal and subsequently converted into an electric pulse. These interactions are recorded over a circular orbit around the patient, followed by digitization and reconstruction into tomographic images [81,82]. The radiopharmaceuticals utilized in SPECT are regional cerebral blood flow compounds, cationic compounds, labelled amino acids, labelled antibodies, labelled somatostatin analogues and apoptosis compounds [83].

Besides the need for radioactive materials, SPECT is characterized by several disadvantages including a low spatial resolution in the case of traditional collimators, which is insufficient for several applications in the human brain. Moreover, the acquisition time of a complete set of projections is up to several minutes [84]. Additionally, the associated infrastructure and instrumentation for this method implies a high cost. Compared to PET, the molecular sensitivity of SPECT is significantly lower. However, considering that the costs are lower, the half-life of the radionuclides used is longer and it uses dual-labelled compounds, this technique is widely applied in clinical practice and preclinical research [85].

#### 2.3.3. Molecular Magnetic Resonance Imaging (mMRI)

The use of contrast agents in MRI has led to the development of a non-invasive method to visualize biological processes at cellular and molecular level. mMRI is used for the detection and localization of disease biomarkers, cells or therapeutic agents [86]. The development of contrast agents is continuously under research, due to the potential to be encapsulated into macromolecular vehicles, such as liposomes or nanoparticles, for pharmacokinetic transport or cells tracking. Additionally, properties related to vascular permeability and perfusion and blood-brain barrier integrity can be determined. Furthermore, the use of mMRI might improve the differentiation between normal and pathological tissues [87]. By attaching or introducing imaging probes into the cells, mMRI can be used for instant assessments of cell-based therapies and as biomarkers for tumour response [88]. Studies have reported the use of various contrast agents for mMRI, especially in cancer research, including perfluoropolyether, iron oxide nanoparticles and microparticles [89], ferumoxide [88], caspase-3-sensitive nanoaggregation MRI [90] and manganese, iron [91] and gadolinium complexes [91,92].

## 3. Nanotechnology-Based Approaches for Neuroimaging

Whereas early detection is highly involved in the efficient treatment of many brain diseases, such as brain cancer [93] and neurodegenerative disorders [94], the development of novel strategies for neuroimaging is crucial [93]. The interlink between nanotechnology and neuroscience, particularly neuroimaging, has shown a great potential in the field of nanomedicine [95], providing new possibilities for designing contrast agents and nanocarriers that target the brain [96]. Nanotechnology-based materials, devices and electronic biosensors allow for enhanced visualization of brain tissue, resulting in greater spatial and temporal resolution and accuracy [95]. Furthermore, nanotechnology approaches improve the identification of biomarkers, the indicators for the biological state of disease, which is of key importance in early diagnosis [93]. Moreover, the blood-brain barrier restricts the permeation of conventional contrast agents that are used for neuroimaging as they are usually hydrophobic in nature and have a reduced half-time circulation. Additionally, large hydrophobic molecules can reach the brain parenchyma mostly through active pathways, such as carrier- and receptor-mediated transcytosis or by disrupting the blood-brain barrier which could lead to serious consequences [94,97]. Therefore, nanotechnology strategies mostly involve the development of nanocarriers that can efficiently reach and permeate the blood-brain barrier after oral or intravenous administration.

**Figure 2:**
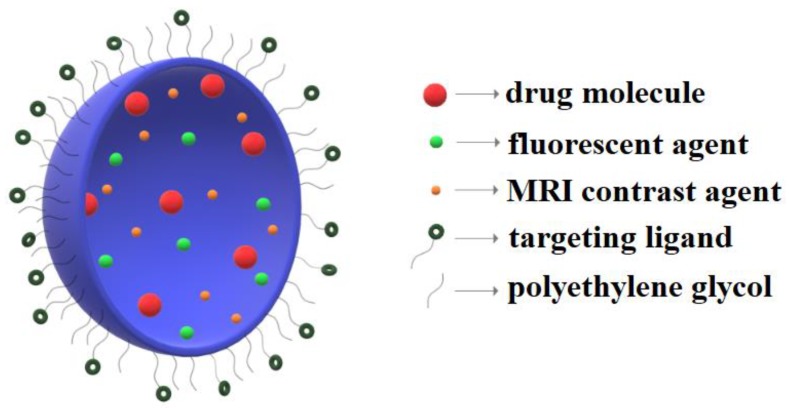
A schematic diagram of the strategies for molecule incorporation and surface functionalization of nanocarriers for neuroimaging.

Additionally, these nanoprobes for neuroimaging offer the possibility of attaching targeting molecules on their surface which could enhance the accumulation at specific sites, such as tumour tissue accumulation (Figure 2).

The main strategies based on nanotechnology and the current associated tests for neuroimaging applications are summarized in Table 2. It should be mentioned that the studies chosen to be included in this review are from the last 5 years, found in databases including but not limited to Scopus and PubMed.

### 3.1. Nanoparticles

The application of nanoparticles has led to the development of potentially novel imaging and diagnostic agents for brain disorders. Nanoparticles can either be used as nanosized imaging agents or as nanocarriers functionalized with contrast agents. Furthermore, they can be designed as theranostic agents through functionalization with therapeutic agents [98]. Thus, the drawbacks of conventional nanoparticles, such as patient compliance and safety, could be overcome by employing theranostic nanoparticles in disease management. [99] Commonly studied imaging agents are iron oxide, gold, manganese oxide and carbon-based nanoparticles.

Iron oxide-based nanoparticles have received outstanding attention as contrast agents owing to their unique physicochemical and superparamagnetic properties. Thus, medical applications, including cell labelling and sorting, cell transfection, diagnostic imaging based on MRI, PET or multimodal imaging could be improved by the use of iron-oxide magnetic nanoparticles [100]. It should be mentioned that ferumoxytol (Feraheme) is a type of magnetic iron oxide nanoparticles which has been approved by the US Food and Drug Administration and it is intensively used in bioimaging [101,102]. In vitro studies reported the use of theranostic iron oxide nanoparticles functionalized with caffeic acid for glioblastoma MRI and reactive oxygen species generation as a therapeutic strategy [103]. Moreover, the theranostic application of iron oxide nanoparticles conjugated with a highly potent vascular disrupting agent and an MMP-14 (matrix metalloproteinase 14) peptide was reported. Results demonstrated their potential for inducing glioblastoma initiating cells apoptosis and impairing tumour growth, as well as the in vivo tracking through MRI [104]. Additionally, iron oxide nanoparticles functionalized with phosphonate polyethylene glycol chains and covalently coupled to cyclic RGD have been applied for in vivo MRI of glioblastoma in mice [105]. The efficiency of these strategies was proved by the preferential accumulation of nanoparticles at the tumour site owing to the passive targeting through the enhanced permeability and retention effect and active targeting induced by cyclic RGD peptides, respectively. The use of superparamagnetic iron oxide nanoparticles has been reported for in vitro and in vivo studies using a gel brain phantom and New Zealand rabbits and a middle-aged human male to rapidly diagnose the emergent stroke through microwave imaging. Injection of the nanoparticles has led to the approximation of an area of reduced attenuation difference associated with ischemic hypo-perfusion of the left carotid circulation [106]. Neuroinflammation could also be diagnosed through a multimodal imaging strategy based on PET and MRI probe as sulphated dextran-coated iron oxide nanoparticles are highly taken by activated microglia [107]. Furthermore, by labelling mesenchymal stem cells with iron oxide nanoparticles, the in vitro process of differentiation into neural-like cells has been visualized through MRI [108].

Gold nanoparticles have attracted a great scientific interest as contrast agents for preoperative, intraoperative and postoperative neuroimaging [109]. Additionally, by attaching chemical moieties and targeting molecules to the surface, gold nanoparticles can be used as multimodal contrast agents with prolonged circulation time, allowing for wider imaging windows [110]. The development of a target-specific imaging system based on peptide-coated gold nanoparticles for specifically detecting glioma cell biomarkers has confirmed the fluorescence signal-based property of the imaging agent [111]. Moreover, gold nanoparticles could permit the visualization of transplanted stem cells inside the brain. One study proposed the use of gadolinium labelled DNA gold nanoparticles for tracking neural stem cells through MRI [112]. In a similar way, CT has been applied for tracking mesenchymal stem cell-derived exosomes through glucose-coated gold nanoparticles [113].

Although all the commercially available intravenous agents contain gadolinium, which is widely studied for MRI, gadolinium-based contrast agents are highly toxic, associated with nephrogenic systemic fibrosis [114,115]. Thus, research scientists have been focusing on developing gadolinium-free contrast agents. Manganese represents the most viable alternative to gadolinium, with the strong paramagnetic properties of high spin Mn^2+^ ion and the long electronic T1 [115]. Manganese oxide nanoparticles were also used for in vivo glioblastoma MRI. Nanoparticles were first synthesized as oleic acid capped nanoparticles and further transformed by replacing the oleic acid with N-(trimethoxysilylpropyl) ethylene diamine triacetic acid silane, which allowed for the subsequent conjugation of folic acid, a glioma-specific moiety [116]. One study focused on the evaluation of the evolution of hypoxic-ischemic brain injury using hollow manganese nanoparticles as positive T1 contrast agents for MRI [117]. The purpose for using hollow nanoparticles is to increase the specific surface area and subsequently the water-surface interactions, which will further enhance the signal contrast [114]. Therefore, the in vivo imaging of the apoptotic brain areas was possible for up to 21 days, which proves the potential of these nanoparticles for monitoring brain injuries [117].

There has been considerable progress made in research works regarding diagnosis and medical imaging based on carbon nanoparticles strategies. For an effective contrast agent development, coupling of carbon nanoparticles with superparamagnetic iron oxide nanoparticles and gadolinium-functionalized carbon nanoparticles represent potential strategies [118]. The conjugation of the Pittsburgh Compound B with carbon nanotubes for imaging Aβ plaque deposition through various techniques might lead to the efficient early diagnosis of Alzheimer’s disease and therapy and disease progression monitoring [119].

Other theranostic approaches for glioblastoma diagnosis and treatment focused on the development of polysiloxane-based nanoparticles, combining a gadolinium-based contrast agent (AGuIX), 5-(4-carboxyphenyl)-10,15,20-triphenylporphyrin as a photosensitizer and the KDKPPR ligand peptide motif targeting neuropilin-1, a receptor overexpressed by angiogenic endothelial cells of the tumour vasculature [120].

### 3.2. Liposomes

Similar to natural cell membranes, liposomes are self-assembled lipid-based bilayer vesicles of varying size and structural complexity [121,122]. Liposomes can incorporate a wide range of polar, non-polar and amphiphilic imaging agents or drugs, both within the aqueous core and the lipid bilayers and target specific sites in the body [121,123].

The application of liposomes for the diagnosis of neurological diseases has been intensively studied and strategies involving the covalent bonding of peptides, antibodies and RNA aptamers and the formulation of external stimuli-responsive liposomes have shown great potential for neuroimaging [124].

Research has reported the application of heptamethine cyanine dye IR780 incorporated into liposomes for in vitro and in vivo near-infrared fluorescence imaging of brain tumours using the human glioblastoma multiforme xenograft model and the spontaneous glioblastoma multiforme mouse model respectively [125]. Furthermore, functionalized polyethylene glycol liposomal formulations with antibodies specific for brain tumours have been studied. Aiming to apply in vivo MRI and optical imaging for non-invasive studies, iron oxide nanoparticles and a near-infrared fluorescence dye were encapsulated in the liposomes. Results showed the efficient uptake of the nanoplatforms at the tumour site, with enhanced spatial and temporal resolution in MRI [126]. The formulation of gadolinium-loaded liposomes functionalized with the GBI-10 aptamer to specifically target tumour cells with the overexpressed Tenascin-C glycoprotein. The in vitro results showed an increased endocytosis of the aptamer-functionalized liposomes and a higher relaxivity compared to commercially available MRI contrast agents [127]. A multi-functional hybrid system containing biocompatible liposomes and magneto-plasmonic nanoparticles for image-guided delivery of anti-HIV drugs to the brain has also been developed. The in vitro distribution of the nanocarriers was assessed through MRI, magnetic particle imaging and micro-CT. Results demonstrated the potential of the multimodal nanosystems for the future development of efficient therapy strategies against HIV infected cells [128].

Another research work reported the administration of liposomes incorporating indocyanine green as cargo and two liposomal markers, 1,2-dipalmitoyl-*sn*-glycero-3-phosphoglycerol and 1,2-distearoyl-*sn*-glycero-3-phosphoethanolamine conjugated with monodisperse polyethylene glycol into the tail vein of male C57BL/6JRj mice [129]. Additionally, paramagnetic chelate gadolinium-diethylenetriaminepentaacetic acid-loaded pH-sensitive liposomes coated with polyethylene glycol were administered for glioma cell targeting and treatment. The targeting and accumulation efficiency of the liposomes was monitored through in vivo MRI [130]. Moreover, a theranostic approach focused on the formulation of liposomes incorporating an anti-cancer drug, doxorubicin and quantum dots for MRI. Results proved that the application of focused ultrasounds improved the glioma-targeted accumulation, as they can reversibly disrupt the blood-brain barrier without damaging the tissues. Thus, this study offers a promising alternative for chemotherapy with minimal side effects in future clinical application [131]. Similarly, RGD-TPGS-functionalized theranostic liposomes containing docetaxel, an anti-cancer drug and quantum dots were developed for brain cancer imaging and treatment. The RGD peptide allows for the active targeting of tumours and for the internalization through receptor-mediated endocytosis. A higher delivery efficiency compared to the commercially available formulation of docetaxel and to the non-targeting theranostic liposomes was proved in the in vivo studies performed on Charles Foster rats [132].

### 3.3. Micelles

Micelles are vesical nanoconstructs formed in aqueous solutions by the self-assembly of amphiphilic molecules, with the hydrophilic/polar region on the external surface and the hydrophobic/non-polar region on the inner surface, forming the core [133]. The usual size of pharmaceutical micellar formulations is less than 80 nanometres and the value of the critical micelle concentration, the concentration at which the monomeric amphiphile forms micelles, should be in the millimolar region [134]. Similar to liposomes, micelles can be conjugated or functionalized with polymers, oligonucleotides, peptides or carbohydrates for improving the specificity and the pharmacological behaviours. However, compared to liposomes, micelles are more rapidly accumulated at the tumour sites owing to their smaller size [135].

Various micellar formulations have been designed as imaging agents for different biomedical imaging techniques, including MRI and CT, for the purposes of brain diseases diagnosis or drug delivery monitoring using micelles as nanocarriers [136].

Hyper-permeable blood-brain barrier areas that could be related to solid tumour tissues have been visualized by using gadolinium-incorporated micelles for MRI. This system represents a novel strategy for quantitative haemorrhage-risk evaluation due to the correlation between the extravasation of micelles and the haemorrhagic oedema site [137]. The early detection of neuroinflammation for investigating the triggering and the progression of neurodegenerative diseases might be possible by using MRI-detectable micelles targeted towards the vascular cell adhesion molecule, which is overexpressed in neuroinflammation. Thus, in vivo injected paramagnetic gadolinium-loaded targeting micelles have the potential to indicate neuroinflammation, making tremendous progress in neuroscience [138].

One study focused on developing a theranostic approach using a novel formulation of gold and superparamagnetic iron oxide-loaded micelles coated with polyethylene glycol and polycaprolactone polymers [139].

### 3.4. Nanobodies

Nanobodies are the antigen-binding or variable heavy chain domain, comprised of four conserved sequence stretches surrounding three hypervariable complementarity-determining regions. Nanobodies are small molecules, possessing unique physical and chemical properties [140]. They are recognized for their solubility, specificity, cost-effective production [141] and remarkable stability under extreme conditions, including high temperature and pressure, low pH and presence of proteases. Furthermore, subsequent to in vivo administration, nanobodies can rapidly diffuse and penetrate body tissues [140]. Therefore, nanobodies represent great candidates for molecular imaging modalities, allowing for fast tumour visualizations owing to homogenous tumour accumulation and fast blood clearance [141].

Moreover, nanobodies can be engineered to detect neuropathological lesions in Alzheimer’s disease, specifically amyloid plaques and neurofibrillary tangles. The in vivo imaging of the nanobodies through two-photon fluorescence imaging (2PFI) represents a fundamental step for unravelling the mechanisms underlying neurodegenerative diseases [142].

### 3.5. Quantum Dots

Defined as ‘small crystals containing a variable number of electrons that occupy well-defined, discrete quantum states and have electronic properties intermediate between bulk and discrete fundamental particle,’ quantum dots represent the new generation of nanosized semiconductor inorganic crystals [143]. Quantum dots possess unique physical and optical properties, including high quantum yield, narrow and tuneable emitting spectra, which make them great candidates as tools in molecular biology, chemical analysis and materials science [144,145].

Furthermore, quantum dots represent a method for unravelling the mechanisms of molecules and cells behaviour inside the body. Marking them with quantum dots could improve in vivo visualization, allowing for tracking them during a specific period of time. Owing to their longer photostability, quantum dots are superior to conventional fluorescence and organic dyes [146].

Carbon quantum dots are widely used in bioimaging applications owing to their excellent biocompatibility, low cost and easy preparation. By functionalization for tissue imaging and brain gliomas targeting, the tumour uptake could be visualized through fluorescence imaging. However, further studies should be developed for the in vivo imaging of the tumour [147]. Recent studies focused on the imaging of polyethylene glycol quantum dots systems at the tumour site through IVIS imaging system. Although targeted accumulations were observed in tumour tissues, the skulls of the mice were removed in order to acquire the images [148].

Similarly, semiconducting polymer dots with donor-acceptor structure were synthesized for the in vivo mouse brain imaging through 2PFI. The in vivo angiography of the brain indicated large penetration depth, demonstrating the potential of 2PFI of polymer dots for deep-tissue in vivo imaging [149].

Another application of quantum dots for neuroimaging is the single-cell in vivo imaging which has evolved with the development of near-infrared fluorophores and nanotechnology strategies for targeted delivery. This technique has the capacity to offer more detailed information for diagnosis purposes [150]. One study reported the use of quantum dots-antibodies conjugates for in vivo cytometry of cells in their unperturbed microenvironment, which could offer information regarding single cell movement and cell-cell and cell-extracellular matrix interactions. These nanosystems are advantageous as they can be easily and efficiently decorated with targeting molecules or secondary reporters, have a low tendency to non-specifically bind to other cells or serum components, exhibit longer blood circulation times, diffuse through dense in vivo environments and have a narrow emission for multiplexed imaging. Moreover, the use of quantum dots as probes for in vivo imaging allow for long-term single cell tracking in healthy or diseased tissues in vivo and for functional analyses, such as oxygen level, glucose concentration or local mechanical stress measurements [151].

**Table 2:** A summary of the nanotechnology-based applications for the diagnosis of brain diseases.

Nanotechnology-Based Strategy	Imaging Probe	Targeting Strategy	Neuroimaging Technique	Targeted Brain Disease	Experimental Stage	Ref.
Iron-oxide nanoparticles	iron oxide nanoparticles functionalized with caffeic acid	passive—enhanced permeability and retention effect	MRI	glioblastoma	in vivo—orthotopic U87-MG tumour implanted in nude mouse brain	[103]
	iron oxide nanoparticles functionalized with phosphonate polyethylene glycol chains and covalently coupled to cyclic RGD	active—cyclic RGD peptides	MRI	glioblastoma	in vivo—orthotopic U87-MG tumour implanted in nude mouse brain	[105]
	superparamagnetic iron oxide nanoparticles	passive	microwave imaging	emergent stroke	in vivo—New Zealand white rabbits; in vivo—middle aged human male volunteer	[106]
	sulphated dextran-coated iron oxide nanoparticles conjugated with the macrocyclic chelator 1,4,7,10-tetraazacyclododecane-1,4,7-triacetic acid	active—sulphated dextran-coating	PET and MRI	neuroinflammation	in vivo—BALB/c mice	[107]
	iron oxide nanoparticles	active—MMP-14 peptide	MRI	glioblastoma	in vivo—NOD scid gamma mice	—
Gold nanoparticles	polyethylene glycol coated gold nanoparticles conjugated with CBP4 peptide	active—CD133 glioma biomarker	laser scanning confocal microscope	glioblastoma	in vivo—U373 glioma cell culture	[111]
Manganese oxide nanoparticles	hollow manganese oxide nanoparticles	passive	MRI	hypoxic-ischemic brain injury	in vivo—Sprague Dawley rat pups	[117]
	N-(trimethoxysilylpropyl) ethylene diamine triacetic acid silane and folic acid-conjugated manganese oxide nanoparticles	active—folic acid, a glioma-specific moiety	MRI	glioblastoma	in vivo—male nude mice (BALB/C)	[116]
Carbon-based nanoparticles	multi-walled carbon nanotubes conjugated with Pittsburgh Compound B and gadolinium complexes	active—Pittsburgh Compound B for binding to Aβ plaques	SPECT and CT	Alzheimer’s disease	in vivo—female C57BL/6 mice	[119]
Polysiloxane-based nanoparticles	AGuIX	active—KDKPPR ligand peptide motif	MRI	glioblastoma	in vivo—dorsal skinfold chamber using female nude mice	[120]
Liposomes	heptamethine cyanine dye IR780 incorporated into liposomes	active—IR780 dye for tumour targeting	near-infrared fluorescence imaging	glioblastoma	in vivo—T98G and U87MG cells; in vivo—nude mice bearing U87M2/luc tumours	[125]
	iron oxide nanoparticles and near-infrared fluorescence dye DiR incorporated into polyethylene glycol liposomes functionalized with the F(ab’)_2_ fragments of PGN635	active—phosphatidylserine targeting	MRI and near-infrared optical imaging	glioblastoma	in vivo—human glioma U87MG cells; in vivo—BALB/c mice	[126]
	paramagnetic chelate gadolinium-diethylenetriaminepentaacetic acid-loaded liposomes coated with polyethylene glycol	passive	MRI	glioblastoma	in vivo—tumour bearing C57BL6 adult male mice	[130]
	gadolinium-loaded liposomes	active—GBI-10 aptamer	MRI	glioblastoma	in vitro—MDA-MB-435s human breast duct cell line	[127]
	quantum dots and doxorubicin-loaded liposomes	active—focused ultrasound	MRI	glioblastoma	in vivo—Adult male Sprague–Dawley rats	[131]
	quantum dots and docetaxel-loaded liposomes	active—RGD-TPGS peptide	—	glioblastoma	in vivo—Charles Foster rats	[132]
Micelles	polyethylene glycol-b-poly(l-lysine-DOTA-gadolinium) micelles	passive—enhanced permeability and retention effect	MRI	ischemia-reperfusion injury	in vivo—Wistar male rats	[137]
	paramagnetic gadolinium-loaded targeting micelles	active—targeting the vascular cell adhesion molecule	MRI	neuroinflammation	in vivo—C57BL/6J female mice	[138]
	gold and superparamagnetic iron oxide-loaded micelles coated with polyethylene glycol and polycaprolactone	Passive	MRI and CT	glioblastoma	in vivo—female athymic nude mice	[139]
Nanobodies	anti-Aβ and anti-pTau VHHs	active—amyloid plaques and neurofibrillary tangles	2PFI	Alzheimer’s disease	in vivo—PS2APP mice overexpressing hAPP Swedish mutation combined with PS2 N141I mutation and Tg4510 mice with the hMAPT P301L gene mutation	[142]
Quantum dots	semiconducting polymer dots encapsulated into poly(styrene-co-maleic anhydride) and conjugated with poly(ethylene glycol)	active—donor-acceptor structure	2PFI	—	in vivo—ICR female mice	[149]

## 4. Conclusions and Perspectives

Conventional neuroimaging techniques possess several disadvantages regarding the spatial and temporal resolution, the invasiveness of the imaging modality, the toxicity of the contrast agents and the costs implicated in image acquiring. As neuroimaging is of key importance in early diagnosis and therapy efficacy monitoring of brain diseases, overcoming the associated limitations is crucial. Nanotechnology represents a potential strategy for improving neuroimaging techniques by developing novel imaging agents or nanocarriers for conventional contrast agents. Although there is a great research performed for designing nanotechnology approaches for brain imaging, further studies are necessary for both in vivo imaging improvement and long-term impact establishment. As there is a limited number of commercially-approved nanocarriers for the targeted delivery of contrast agents to the brain, thorough investigations regarding their toxicity and their associated side effects, as well as the ways to overcome these limitations must be performed.

As future perspectives, specialists in the field of neuroimaging should consider the application of nanotechnology for single-neuron detection [152,153] and phase-contrast X-ray imaging techniques [154] which could offer the possibility of visualizing brain areas and activities that are currently obscure or undetectable for conventional methods. In the case of single-neuron detection, current studies focus on the use of diamond chips with quantum defects as sensors that can detect time-varying magnetic fields generated by action potentials of neurons [155]. Similarly, magnetic induction tomography performed with optical atomic magnetometry allows for the detection across a large frequency range in low-conductivity targets, such as the biological tissues [156]. Moreover, the phase-contrast X-ray imaging offers the possibility of 3D visualization of soft tissue-organs, such as the lung and brain. The currently available techniques that use phase-contrast are propagation- and analyser-based, crystal and grafting interferometry and non-interferometry methods [157]. This technique might overcome the poor soft tissue contrast of conventional CT and the poor spatial resolution of MRI [158].

## Figures and Tables

**Table 1:** A summary of the main characteristics of the neuroimaging techniques previously presented.

	CT	MRI	fMRI	EEG	iEEG	fNIRS	fUS	PET	SPECT	mMRI
Cost	$–$$	$$	—	$	—	$–$$	—	$$$	$$–$$$	—
Invasiveness	minimal	non-invasive	non-invasive	non-invasive	requires surgery	non-invasive	non-invasive	minimal	minimal	non-invasive
Acquisition time	minutes	minutes to hours	minutes to hours	minutes	hours	minutes	—	minutes	minutes	minutes to hours
Portability	not portable	not portable	not portable	portable	not portable	portable	portable	not portable	not portable	not portable
Personnel requirements	qualified personnel	qualified personnel	qualified personnel	qualified personnel—optional	qualified personnel	qualified personnel—optional	qualified personnel—optional	qualified personnel	qualified personnel	qualified personnel
Spatial resolution	0.5–0.625 mm	1–2 mm	1–2 mm	5–9 cm	4.5 mm	1 cm	50–200 µm	3–5 mm	6–8 mm	—
Temporal resolution	85–135 ms	20–50 ms	1–3 s	130 ms	0.8 ms	330 ms	1–100 ms	5 s to 5 min	15 min	—
Penetration depth	no limit	no limit	1.2 mm	—	—	3 cm	no limit	no limit	no limit	—
